# StratosPHere 2: statistical analysis plan for a response-adaptive randomised placebo-controlled phase II trial to evaluate hydroxychloroquine and phenylbutyrate in pulmonary arterial hypertension caused by mutations in BMPR2

**DOI:** 10.1186/s13063-025-08947-y

**Published:** 2025-07-11

**Authors:** Rajenki Das, Nina Deliu, Mark R. Toshner, Sofía S. Villar

**Affiliations:** 1 MRC Biostatistics Unit, University of Cambridge, Cambridge, UK; 2 MEMOTEF, Sapienza University of Rome, Rome, Italy; 3 Victor Phillip Dahdaleh Heart & Lung Research Institute, University of Cambridge, Cambridge, UK; 4 Royal Papworth Hospital, Cambridge, UK

## Abstract

**Background:**

The StratosPHere 2 trial will evaluate the efficacy of hydroxychloroquine and phenylbutyrate in pulmonary arterial hypertension caused by mutations in BMPR2 by focussing on the novel biomarker and other endpoints including safety.

**Study design:**

StratosPHere 2 is a three armed, placebo-controlled, phase 2 trial with two strata based on the mutation groups. It is response adaptive where the allocation of treatments follows a Bayesian response-adaptive randomisation algorithm. An expected number of 20 patients will be randomised in each stratum to one of the three arms containing hydroxychloroquine, phenylbutyrate and placebo. The primary outcome is a novel endpoint considering the change in the bone morphogenetic receptor type 2 (BMPR2).

**Method:**

The final primary analysis on the efficacy of each active treatment against control is assessed using a one-sided nonparametric Wilcoxon test computed on the continuous biomarker data collected up to 8 weeks from the start of treatment.

**Discussion:**

This manuscript presents the key elements of the StratosPHere 2 implementation and statistical analysis plan. This is submitted to the journal before the first interim analysis to preserve the scientific integrity under a response-adaptive design framework.

The StratosPHere 2 trial closely follows published guidelines for the content of Statistical Analysis Plans in clinical trials.

**Trial registration:**

The ISRCTN Registry ISRCTN10304915 (22/09/2023)

## Background

Pulmonary arterial hypertension (PAH) is a life-threatening disease where increased resistance in the pulmonary arteries leads to heart failure and death if untreated. Patients, often diagnosed young, face life-long invasive and costly treatments, with no current cure. In the UK, the 5-year survival from diagnosis is 56% for idiopathic, heritable or drug-induced PAH without comorbidities [1]. Current therapies address symptoms, not the underlying cause, with high costs: oral therapy QALY costs range from C$146,254 to C$412,979 [2], and IV therapy costs £343,000/QALY [3].

Mutations in the BMPR2 protein cause 29% of idiopathic and familial PAH, with BMPR2 dysfunction implicated in other forms. No current therapies target the BMPR2 pathway, even though data from a recent phase II study in modulating negative regulators of the pathway using a transforming growth factor ligand trap (sotatercept) have demonstrated proof of concept, and the unpublished phase III study in idiopathic PAH (NCT04576988) has been reported [4] as significantly achieving its primary endpoint. Preclinical studies suggest hydroxychloroquine prevents lysosomal BMPR2 degradation [5, 6], and phenylbutyrate reduces ER stress and misfolding, reversing PAH in murine models [7].

StratosPHere 2 will test the hypothesis that BMPR2 dysfunction can be reversed using the preclinically validated and repurposed therapies (hydroxychloroquine and phenybutyrate) therapies in a randomised, placebo-controlled trial using a response-adaptive design to optimise resource use in a small population. This study builds on StratosPHere 1 which defined biomarkers for BMPR2 target engagement, and aims to establish the potential of these therapies for disease modification.

This paper outlines the statistical analysis plan for the StratosPHere 2 trial at interim and final stages of the trial based on the Protocol [8]. It also includes implementation strategies which have been adopted to make the trial more efficient, which are most relevant from a design point of view, i.e. when the trial is ongoing.

The trial Protocol has already been published in *Trials* [8]. The trial is being conducted in compliance with the Protocol, GCP and the applicable regulatory requirements.

### Objectives

#### Primary objectives

Target engagement as defined by the demonstration of BMPR2 function using a panel of qPCR of BMPR2 target genes in peripheral blood. Target engagement will be quantified in terms of a combination of the individual changes in 8 qPCR from baseline (study entry; prior to any treatment) to follow-up (8 weeks from treatment initiation) in the primary endpoint (specified in Primary outcomes in Protocol), independently for each of the two mutation strata and for each of the treatments considered. The 8-week follow-up timeframe for the primary endpoint is dictated by pharmacokinetics aspects: hydroxychloroquine has a half-life of 30–50 days which needs to be considered in the response-adaptive design so that interim data can be expected to be informative enough of both experimental arms.

#### Secondary objectives

 

- Evaluating the effectiveness of any of the two repurposed therapies in the pooled trial sample, independently on the substratum by functional assessment (6MWT) and cardiac functional assessment (NT-proBNP).
- Exploration of efficacy in terms of patient-related outcomes and function (EmPHasis 10).
- Exploratory additional secondary measurements of BMPR2 expression and function. These analysis would inform the definition of primary and secondary outcomes for studies exploring similar mechanistic hypotheses regarding target engagement of BMPR2 function.

## Study design and methods

### Study design

**Fig. 1:**
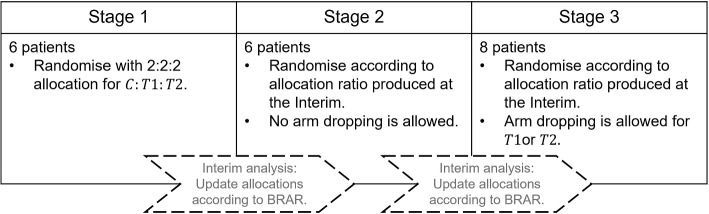
Schematic of the *StratosPHere 2* trial design for one stratum

StratosPHere 2 is a response-adaptive randomised controlled trial and is stratified according to two mutation sub-classes, and considers two active arms (*T*1 and *T*2) and a control arm (*C*). The overall trial design is characterised by recruiting patients into three cohorts (or stages) each of sizes 6, 6 and 8 respectively, per each stratum (see Fig. 1). Two interim analyses, performed between each of the two consecutive stages per stratum, are planned. At each interim, the allocation probabilities are updated independently for each mutation class using a Bayesian response-adaptive randomisation (BRAR) algorithm described in Bayesian response-adaptive randomisation section. For further details, we refer to the Study Protocol [8]. This Statistical Analysis Plan (SAP) also outlines the practical implementation of the BRAR design through a *Mapping* strategy, which is discussed in Mapping to target allocation ratio section.

The design choice of (6,6,8) was made taking into account recruitment considerations in a rare disease setting in conjunction with the feasibility of implementing the BRAR design and simulation results on the design’s operating characteristics. The sizes of the first two stages are chosen to allow for a fair allocation ratio in case of similar treatment responses. In particular, stage 1 implements restricted randomisation with an allocation ratio of 2:2:2, making the choice of 6 an ideal solution. Stage 2 is allowed to have a less balanced allocation ration according to the BRAR rule. For the last stage, we empirically compared the final choice of (6,6,8) with a more sequential option of (6,6,4,4) for the sample sample size of $n=20$. Based on 10,000 replicas, the two showed similar results in terms of type I error and power, and a slightly improved allocation of the superior arm with (6,6,8). Therefore, this was preferred, given also its lower demand in terms of the number of interim analyses.

#### Bayesian response-adaptive randomisation

We use the BRAR algorithm of [9, 10]. This rule defines randomisation probabilities at each interim analysis based on the accumulated response data up to that point of the trial and ensures control protection using a tuning parameter $\eta _t$ to guarantee at least 1/*K* allocation to the control arm.

Formally, let $\mathcal {D}_{n_{\overline{t-1}, s}}$ denote the observed data (assigned arms and responses) from $n_{\overline{t-1},s}$ participants in stratum *s* across all prior stages $\overline{t-1} = \{1, \dots , t-1\}$. For a given trial stage *t*, stratum *s* and treatment arm *k*, the allocation probabilities $\pi _{t, s, k}$ are given as:

$$\begin{aligned} \pi _{t, s, k} (\gamma _t, \eta _t) \propto \left\{ \begin{array}{ll} \frac{p(\theta _k> \theta _C \mid \mathcal {D}_{n_{\overline{t-1}, s}})^{\gamma _t}}{\sum _{j \in \{T1, T2\}} p(\theta _j> \theta _C \mid \mathcal {D}_{n_{\overline{t-1}, s}})^{\gamma _t}} & \text {if}\ k = T1, T2, \\ \frac{1}{K}\exp \left( \max (n_{t-1, s, T1}, n_{t-1, s, T2}) - n_{t-1, s, C}\right) ^{\eta _t} & \text {if}\ k = C, \end{array}\right. \end{aligned}$$

where $p(\theta _k> \theta _C \mid \mathcal {D}_{n_{\overline{t-1}, s}})$ is the posterior probability that arm *k* is better than control and $\gamma _t$ and $\eta _t$ are tuning parameters controlling imbalance between the active arm and ensuring minimum allocation to the control arm respectively. $n_{t-1, s, k}$ is the number of participants assigned to arm *k* in stratum *s* up to stage $t-1$. Due to the novelty of the stufy, we assume “non-informative” Beta(1, 1) prior distributions on the unknown parameters $\theta _k$ for $k = C, T1, T2$.

### Blinding

The trial has a *veiled* [11] blinding (i.e. patients and doctors are blinded to active vs. placebo, but not treatment type) as reported in the Study Protocol, and further discussed in Level of blinding section of this Statistical Analysis Plan (SAP).

### Study outcomes

#### Primary outcomes

The primary outcome is a measure of target engagement of the BMPR2 pathway, defined by the change in peripheral blood-based BMPR2 function, denoted as $\Delta BMPR2$ from baseline (study entry) to follow-up at 8 weeks. Thus, the primary endpoint is computed as:

$$\begin{aligned} \Delta BMPR2_s^F & = \frac{1}{8} \left( |\Delta {ID3}_s^F| + |\Delta {SMAD1}_s^F| + |\Delta {SMAD5}_s^F| + |\Delta {NOTCH1}_s^F| + \right. \\ & \qquad \left. |\Delta {NOTCH2}_s^F| + |\Delta {ID2}_s^F| + |\Delta {ARL4C}_s^F| + |\Delta {PTGS2}_s^F| \right) , \quad s = A, B. \end{aligned}$$

Here, *s* is the substratum corresponding to the mutation group (A: haploinsufficient, B: missense) and $\Delta Y_s^F$ is the change in gene expression of the biomarker of interest. For a generic qPCR gene product $Y^s$, $\Delta Y_s^F$ is defined as:

$$\begin{aligned} \Delta Y_s^F = \left| 2^{-\Delta \Delta \text {Ct}(Y^s)_F} - 1 \right| , \end{aligned}$$

where $\Delta Ct(Y^s_t) = Ct({Y^s}_t) - Ct({HK^s}_t)$ for $t = 0,F$, where *HK* is the housekeeping gene and *Ct* is the cycle threshold of the sample. $\Delta Ct$ normalises *Y* relative to *HK* to account for experimental variability. The follow-up *F* is considered as 8 weeks from baseline. This endpoint will be used for both the study’s final primary analysis and as an adaptation endpoint for informing allocation of subsequent trial participants and potentially dropping of an arm.

##### Adaptation endpoint

During the interim, the adaptation endpoint considered is the binary variable having value 1 (success) if the arm shows a change of at least 30% in the primary endpoint, and 0 (failure), otherwise. We emphasise that this (binary) adaptation endpoint is meant to guide the response-adaptive design in order to determine the allocation probabilities and will be computed during the interim analyses only. The continuous version of this outcome is used during the final analyses when the hypotheses will be tested.

#### Safety and secondary outcomes

Secondary analyses will evaluate the following outcomes:

1. Safety: Incidence and severity of adverse events, as defined in Safety reports section
2. Functional, efficacy and quality of life measures:
  $\Delta {BMPR2}^s_F$ from baseline to follow-up at $F = 16$ and $F = 20$ weeks, for each stratum $s \in \{A, B\}$
  6-min walk test
  NT-proBNP levels
  Health-related quality of life (HRQoL) measured by EmPHasis10
3. Biomarkers:
  BMPR2 cell surface protein expression on peripheral blood white cells
  Change in individual qPCR biomarkers from the primary endpoint panel
  Change in RNAseq peripheral blood expression

### Sample size determination

A sample size of 20 patients per stratum represents a realistic patient accrual for this trial. This sample size was evaluated in simulation studies for superiority hypothesis testing under an effect size of $30\%$ genetic pathway variation from baseline to 8-week follow-up. Satisfactory results are obtained in terms of both frequentist operating characteristics and patient allocation to the optimal treatment. A one-sided nonparametric Wilcoxon test was used, giving 80% power under a 10% type I error control with $n=20$; we refer to the Study Protocol [8] for further details. The results remain satisfactory once a *Mapping* strategy (see Mapping to target allocation ratio section) is used to safely implement the BRAR procedure in practice; simulation results are provided in Table 3 in Appendix D.

## Analysis types, timing and cohorts

The primary analysis for this study will follow the ‘intention to treat’ (ITT) approach including all randomised subjects regardless of whether they received the treatment randomly allocated to them or completed follow-up. Data will be analysed assuming each patient received their assigned treatment. Further eligibility criteria are provided in the Protocol. For the interim analysis, all randomised subjects with the 8-week follow-up primary endpoint will be considered. In case of missing data, the missing data mechanisms specified in the SAP will be followed.

A ‘per protocol’ analysis will be conducted if sufficient data on non-adherence is available, and the extent of bias will be discussed at the final analysis. The per-protocol population includes subjects who completed the study without major protocol deviations. This analysis will serve as a secondary, sensitivity analysis.

Here we list the types and timelines of analyses to be conducted in the StratosPHere2 trial, along with the associated cohorts. Three types of analyses will be performed—interim (during the course of the trial), final (at the conclusion of each stratum) and pooled (after completion of study for both strata). Safety reports are provided at the time of each of these analyses. All analyses will apply to data collected during the trial and will be conducted using R [12]. The trial is stratified based on the mutation class of participants, and the analysis cohorts are defined accordingly.

- Interim analyses: two interim analyses will be conducted for each stratum at the end of each stage according to the trial design:
  - First interim analysis: will happen after the first six patients in a stratum are randomised and followed up. Once their biomarker data are available, the interim report on adaptation and a safety report will be prepared at this stage.
  - Second interim analysis: will happen after the completion of second stage of the trial, i.e. after the first 12 patients in the stratum are randomised.
- Final analyses: for each stratum of the trial, the final analysis will be conducted at the end of third stage of the trial when all the patients who are in the study have completed their studies and their biomarker data are available. Therefore, the cohort contains all subjects who were randomised in the stratum.
- Pooled analyses: this will be performed once data from both the strata are available. Cohort is of all patients who were randomised.
- Safety reporting: this takes place at the same time as each of the above analyses, considering the corresponding analysis population.

## Implementation plan

### Mapping to target allocation ratio

An intermediate step of *Mapping* is introduced to the trial design for the implementation of randomisation. This step helps generate the randomisation sequence for the next stage by mapping the continuous randomisation probabilities derived at the interim to a discrete allocation ratios using a decision rule as detailed in [13].

### Updating randomisation sequence

**Fig. 2:**
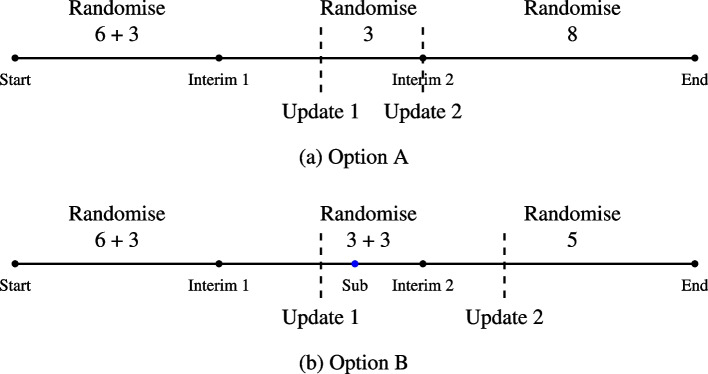
Timeline of randomisation sequence updates following adaptations at interims points. **a** Option A shows the basic timeline while, **b** option B shows a modified timeline allowing for a sub-interim (Sub)

The trial utilises Sealed Envelope Ltd. [14] to implement randomisation of patients to the treatment groups. This requires the unblinded statistician to upload the randomised sequence to the system. To improve the efficiency of randomisation by allowing the trial to continue recruitment of patients without pausing for the first interim analysis, we follow these steps describing the schematic of the timeline of randomisation in option A of Fig. 2:

1. At the start of the trial, we upload one complete randomisation sequence with balanced allocation across all three stages. If there is no adaptation, this approach ensures continuity of randomisation to the treatments without any interruptions. In the case of adaptation, we can still continue the recruitment till patient 9 as originally planned—at this point, the new randomisation will be uploaded if an adaptation is triggered after a discussion with the IDMC. Thus, this saves time and lowers dependency on the unblinded statistician and other people to upload a new sequence.
2. During the second interim of the stratum, the interim and the update points coincide, i.e. we stop the recruitment here for the interim analysis, and in the case of adaptation, the new sequence is uploaded to Sealed Envelope at the start of stage 3.

#### Sub-interim

However, in order to avoid an unnecessary pause in the final leg of recruitment, we may carry out an analysis after at least 4 patients are randomised and have completed their 8 weeks of treatment in stage 2. This analysis is just to assess the probability of dropping ($p_{drop}$) an active arm in the third (final) stage of the study as shown in option B of Fig. 2.

Thus, utilising this information, we have the following decision rule:

1. If $p_{drop}> 0$, pause the recruitment for the second interim as planned, i.e. after 6 patients in stage 2 have completed 8 weeks of treatment.
2. If $p_{drop} = 0$, no need to pause the recruitment for the second interim. We may pause the recruitment after the first 3 patients have completed their 8 weeks of treatment in stage 3. These first 3 patients will be allocated in the ratio of 1:1:1 to the treatment groups $C : T_1 : T_2$. Therefore, update 2 might happen after 3 patients of stage 3.

This assessment will be done by the blinded statistician to preserve integrity of the trial. No information beyond planned at interim 3 will be released as a consequence of this procedure. All that may be learnt is that an arm is or not dropped but this was planned to be disclosed at that stage in any case.

## Interim analysis

### Generating randomisation sequence

We follow the steps of *Mapping* (Mapping to target allocation ratio section) to finalise the allocation ratio using which the randomisation sequence of treatments will be generated and uploaded to Sealed Envelope system. We follow the timeline of Updating randomisation sequence section to update sequences. This will be carried out by the unblinded statistician. Generation of the randomisation sequences has been stored internally with the trial sponsors and can be viewed by only those who are unblinded during the study. The full details can be disclosed at the end of the study.

### Handling missing data

This section describes how complete missingness, defined as complete absence of all biomarker data and thus, the primary endpoint of a patient. This can happen due to various reasons such as the sites may fail to fill the tubes properly, a patient has dropped out of the study and does not have a follow-up blood test or want their data to be excluded from the study. Keeping these in mind, we consider the following cases of missing data where we consider at most 2 patients’ data on the primary endpoint are completely missing.

- Case 1: One participant missing in stage 1; resulting composition: (5,6,8).
- Case 2: Two participants missing in stage 1; resulting composition: (4,6,8).
- Case 3: One participant missing in stage 2; resulting composition: (6,5,8).
- Case 4: Two participants missing in stage 2; resulting composition: (6,4,8).
- Case 5: One participant missing in each of stages 1 and 2; resulting composition: (5,5,8).

Here, we have not considered partial missingness, i.e. a person may have incomplete or partial complete information on biomarker panel. This scenario is highly unlikely because, especially based on StratosPHere 1, it is not possible to miss a datapoint for a biomarker due to biological reasons, and in case of a technical error, the assay can be repeated. This is how we address the above missing data scenarios:

- If any of the first 6 primary endpoint outcomes is completely missing during the first interim (cases 1, 2 and 5), stage 2 proceeds with balanced allocation (2:2:2).
- If any of the second 6 primary endpoint outcomes is completely missing (cases 3, 4 and 5), no treatment arm will be dropped but favouring an arm is still accepted if supported by the data.

Given the small sample size and the complex decision required regarding imputation methodology (statistical or expert), interim missing data points are not imputed.

### Safety reporting

Composition of adverse events, crude rate and incidence proportion at week 8, and a diagram will be presented, as defined in Safety reports section.

### Early stopping for futility

The full trial will only be stopped if there are any safety concerns. Additionally, a treatment arm can be stopped based on the recommendations from the adaptive design of this study. In such cases, the stopping criteria would be due to the pre-defined futility rule described below.

In the worst-case scenarios of observing: (1) 2 successes in *C* and 0 in *T*1 and *T*2, with 0 failures in *C* and 2 each in *T*1 and *T*2 by stage 1; (2) 4 successes in *C* and 0 in *T*1 and *T*2, with 0 failures in *C* and 4 each in *T*1 and *T*2 by stage 2; or (3) 3 successes in *C* and 0 in *T*1 and *T*2, with 1 failure in *C* and 4 each in *T*1 and *T*2 by stage 2, then we would recommend to the Data Monitoring and Ethics Committee (DMEC) that early stopping due to futility be discussed. This would require reassessing the final analysis endpoint instead of the adaptation endpoint to inform the decision of stopping. This is recommended because in these worst cases, we see very little evidence of a treatment effect for the binary endpoint, i.e. the possibility of an active arm being more effective than the control arm if we continue the trial is low, thus, leading to futility.

### Data reporting and monitoring

Interim analyses will occur at two pre-specified time points as illustrated in the schematic of the StratosPHere 2 trial design. For each mutation stratum independently, two interim analyses will be carried out based on the observed primary outcome data at 8 weeks for that stratum only, to inform the allocation probabilities at subsequent stages. Interim analyses will be performed on the interim analysis population: all those patients that were randomised and for which the primary (8-week follow-up) endpoint was observed. To perform each of the four (two for each stratum) interim analyses, 6 patients are expected to be enrolled and randomised in each stratum’s block. To perform each of the two (two for each stratum) final primary analyses, we expect 8 patients in each stratum’s final blocks. Interim analyses will be conducted by the unblinded statistician, while the blinded statisticians will be involved in the final analyses. An Independent Data Monitoring Committee (IDMC) will review safety and endorse an adaptation triggered when appropriate (for example, whether to favour the treatment arm or not, or possibly drop an arm if needed, and the possibility of terminating the trial earlier), and a Trial Steering Committee (TSC) will oversee the implementation of trial recommendations coming from the IDMC. At the interims, the following information will be reported to the IDMC:

- Safety reports
- Demographics data at baseline: age, sex, ethnicity
- Observation data at baseline: height, weight, body surface area, WHO class
- Mutation group of a patient: HAP or MISS
- Baseline biomarker data: ID3, SMAD1, SMAD5, NOTCH1, NOTCH2, ID2, ARL4C, PTGS2
- Follow-up biomarker data: ID3, SMAD1, SMAD5, NOTCH1, NOTCH2, ID2, ARL4C, PTGS2
- Primary outcome, i.e. $$\Delta {BMPR2}^s_F$$
- Success of treatment per arm
- Probability thresholds p* for Mapping
- Adaptation recommended by the design, if any
- Allocation ratio for next stage

At this stage, a decision will be made whether to adapt which will be shared with the IDMC during the closed session. Two examples are provided in the Appendix. Interim analyses will be scheduled at regular calendar time intervals, allowing flexibility to incorporate external information. IDMC meetings will occur regularly, with the ability to amend the frequency of meetings. Interim analysis will focus solely on reporting safety and determining the allocation ratios. No additional analysis will be performed at these stages.

## Final analysis

### Primary analysis

In this trial, the primary objective is to assess the efficacy of the two active treatments as detailed in the Protocol. Here it is assumed that *T*1 is effective, if at all, for the mutation stratum $B =$ ‘haploinsufficiency’ while *T*2 works for the stratum $A =$ ‘missense’. Thus, the most likely scenario which we refer to as case I is represented by the following set of hypotheses null $H_0$ and $H_1$ which are tested using a one-sided non-parametric Wilcoxon test:

Stratum A ‘missense’:

$$\begin{aligned} H_0^A = E(\Delta BMPR2^A_F)_{T2} - E(\Delta BMPR2^A_F)_C \le 0, \end{aligned}$$

$$\begin{aligned} H_1^A = E(\Delta BMPR2^A_F)_{T2} - E(\Delta BMPR2^A_F)_C> 0 \end{aligned}$$

Stratum B ‘haploinsufficiency’:

$$\begin{aligned} H_0^B = E(\Delta BMPR2^B_F)_{T1} - E(\Delta BMPR2^B_F)_C \le 0, \end{aligned}$$

$$\begin{aligned} H_1^B = E(\Delta BMPR2^B_F)_{T1} - E(\Delta BMPR2^B_F)_C> 0 \end{aligned}$$

Case II is an extended analysis where the following set of four hypotheses are tested for $s = A, B; k = T1, T2$:

$$\begin{aligned} H^s_0 = E({\Delta BMPR2^s}_F)_{k} - E({\Delta BMPR2^s}_F)_C \le 0, \end{aligned}$$

$$\begin{aligned} H^s_1 = E({\Delta BMPR2^s}_F)_{k} - E({\Delta BMPR2^s}_F)_C> 0 \end{aligned}$$

### Secondary analysis

#### Estimating treatment effects

The average treatment effect (ATE), defined as $ATE^s_{k} = E(\Delta BMPR2^s_F)_{k} - E(\Delta BMPR2^s_F)_C$, will be estimated using the inverse-probability weighted (IPW) estimator, for each stratum $s = A, B$ and for each active treatment $k = T1, T2$. Compared to the maximum likelihood estimator, the IPW approach ensures unbiasedness in response-adaptive designs such as the BRAR considered in StratosPHere 2 [15]. Multiple testing is adjusted using the Bonferroni correction.

#### Pooled data analysis

A pooled final hypothesis testing analysis, combining data across strata, is preplanned by protocol as a secondary analysis. Given the intrinsic difference between the nature of the two strata and the related disease pathway, we anticipate that such a pooled analysis will only be performed when active treatments show a similar mechanism of action in the two strata. With a similar mechanism, we refer to a similar direction of the estimated treatment effect, formally defined by its sign. Specifically, denoted by $\widehat{ATE}^A_{k}$ the estimated ATE, the pooled analysis will be carried out if $\text {sign}(\widehat{ATE}^A_{k}) = \text {sign}(\widehat{ATE}^B_{k})$ for each $k = T1, T2$. The decision rule is based on simulation studies in which we have evaluated the operating characteristics (type I error and power) of a *stand-alone* analysis (i.e. independently by stratum, each based on $n=20$) *versus* a pooled analysis (based on $N=40$). We refer to Appendix G for details. The IPW estimator will be used to estimate the ATE. The pooled data analysis will also account for multiple testing adjustments, given the two active treatments that are being evaluated. The pairwise Wilcoxon test with the Bonferroni correction method will be used for this purpose.

#### Level of blinding

The level of blinding in this trial is *veiled* [11] where patients receive one treatment from the set *T*1, *T*2, *C*1, *C*2 containing the two active treatments *T*1 and *T*2 and two control arms *C*1 and *C*2 corresponding to the two forms of treatments (tablet and liquid). This still ensures the unpredictability of the arm—whether active or placebo, but it could be inferred which active treatment has not been given to them. This approach helps reduce the cost and patient burden while maintaining sufficient blinding for minimising bias.

The statistical implication of the blinding gets reflected in the hypothesis testing. As under veiled blinding, we compare *T*1 and *T*2 with *C* and not with *C*1 and *C*2. Had it been a double-blinded study, we would have compare *T*1 and *T*2 with *C*1 and *C*2 respectively and in this case, the operating characteristics would have suffered as the power would have dropped to less than 50%. The effect of blinding can be analysed again in the end.

#### Covariates and subgroups

No formal subgroup analysis will be conducted beyond the strata. Anything else is purely descriptive.

### Missing data at the final analysis

As mentioned in Handling missing data section, we expect only complete missingness, if any. However by the end of the trial, more missing cases may arise other than the ones already listed.

Missingness will be reported in terms of missing rates along with the details of the treatment arm, mutation group, time-point in the trial, reason and demographics. If there are no safety concerns and the missingness is upto 2 persons in a stratum, then assuming missingness completely at random, we will continue with the primary and other analyses by considering the complete data. We will not impute because of the small sample size and the rare nature of the disease.

### Other analyses

Summary of the study data will be presented as descriptive statistics. Efficacy and treatment effects at longer time points will be analysed with descriptive statistics.

## Safety reports

Safety is monitored continuously but if any adverse event (AE) occurs, it must be reported immediately to the PI and subsequently to the DMEC during the scheduled meetings. Individual adverse events/adverse reactions should be evaluated by the investigator including the assessment of their seriousness, causality, and any relationship between the investigational medicinal product(s) and/or concomitant therapy and the adverse event. If an AE is determined, expedited reporting is not required. All adverse events should be recorded in the trial eCRF. For every adverse event, the start and end dates will be stored.

Tables will be presented with the columns of adverse events and the treatment arms. There will not be any further bifurcation between the two types of control (liquid and tablet) administered. The following will be reported for each AE *i*. Incidence proportion at a given time point accounts for the differential duration of treatments and follow-up [16].

- AE_*i*_ composition: $\frac{\#AE_i}{\sum \#AE}$, where the numerator is the number of occurrences of $AE_i$ and denominator represents the total number of adverse events.
- Crude rate of AE_*i*_: $\frac{\# \text {pts experienced}\ AE_i\ \text {at least once}}{\sum \#pts}$ which represents the proportion of patients (pts) who have experienced an AE$_i$ at least once.
- Incidence proportion at a given time point $u\in\lbrack0,t\rbrack$: $\frac{\sum _{u \le t} \text {pts experienced}\ AE_i\ \text {at least once}}{\sum \#pts}$, where the numerator is the number of patients experiencing at least one AE at a specific time point *u* within time *t*. The selected time point point for the first interim analysis is 8 weeks.
- Timeline plot: A timeline plot will be presented showing the first occurrences of different adverse events during the duration of study for each patient. Only the System Organ Class (SOC) will be reported.

Same reports will be presented for serious adverse events (SAEs) as well.

## Trial status and discussion

The trial is ongoing. The first and the sixth patients of the same stratum were recruited on 8th March 2024 and 21st January 2025 respectively. The first interim meeting with the Independent Data Monitoring Committee Meeting took place on 6th May 2025. Ninth patient of the stratum was recruited on 25th May 2025.

To maintain the scientific integrity of an adaptive design trial, this SAP was submitted to the journal on 3rd April 2025 which was before the first interim. An extended version of the SAP is being stored internally with the sponsors.

Please refer to the Table 1 in Appendix A for the version history of the SAP stored internally with the trial sponsor.

## Figures and Tables

**Fig. 3:**
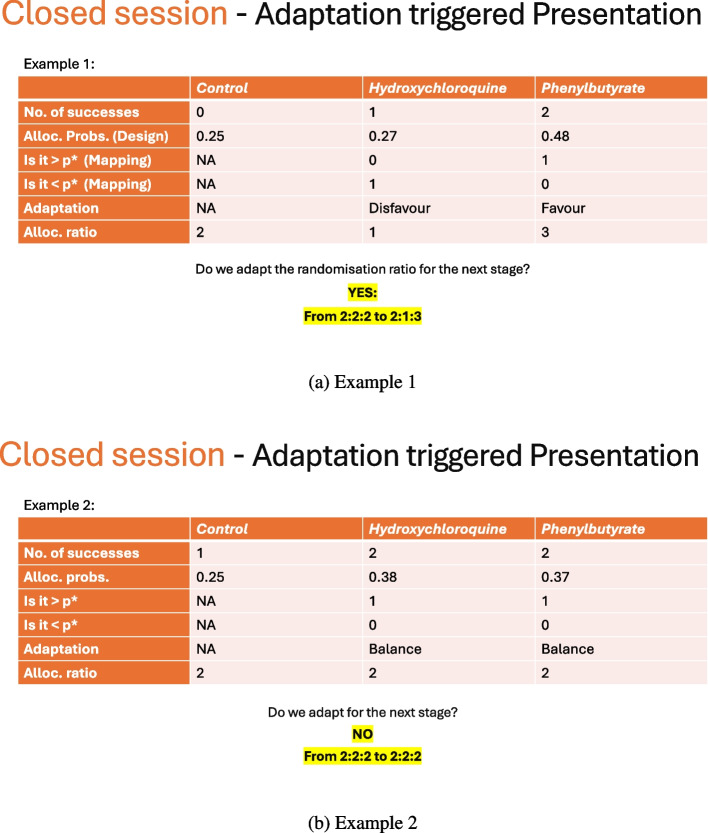
Examples of closed session reports to be presented to the IDMC

**Fig. 4:**
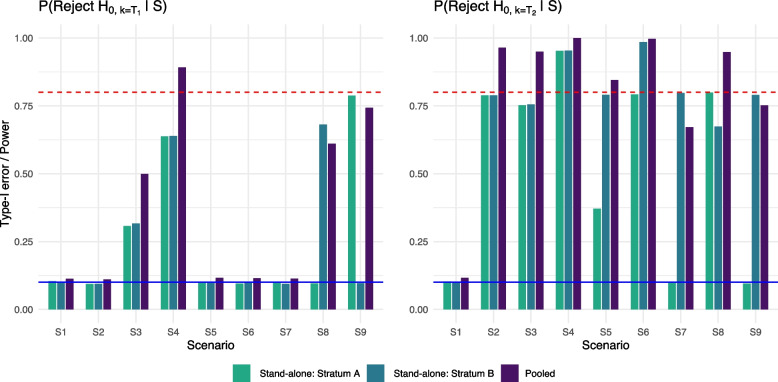
Type I error and power (average results across 10,000 replicas) achieved by a stand-alone analysis by stratum ($n=20$) vs. a pooled analysis ($N=40$) in the evaluated scenarios. The horizontal lines indicate the targeted type 1 error ($\alpha = 0.1$; solid blue line) and power ($1-\beta = 0.8$; dashed red line)

## Data Availability

Ownership of the data arising from this trial resides with the study team. The results of StratosPHere 2 will be made publicly available following funder (MRC) approval. A separate Publication Policy document will be reported in SAP and the data supporting the findings of this study will be available from the authors upon request.

## Appendix A: SAP version history

The version details provided are in accordance to the longer version of the SAP stored with the sponsors. Here the major changes to the SAP are listed.

**Table 1:** SAP version history

Version, date	Revision	Justification
v1.0, 25/3/2025	–	–
v2.0, 18/6/2025	(i) Addition of reference (Hoeper, 2023) in Background and (ii) addition of sub-interim	(i) As asked by reviewer of the journal *Trials* and (ii) to delay or eliminate the pause in recruitment for the second interim analysis.

## Appendix B: IDMC report example

Two examples representing IDMC reporting in a closed session are given in Fig. 3.

## Appendix C: Futility rule

To check for futility, we perform simulations and calculate the treatment effects under different scenarios as presented in Table 2:

**Table 2:** Treatment effects under different scenarios of treatment outcomes

Case	Upto stage	#Success in *C*, *T*1, *T*2	#Failures in *C*, *T*1, *T*2	$\varvec{P(T1)> C}$	$\varvec{P(T2)> C}$
1	1	0, 0, 0	2, 2, 2	0.5	0.5
2	1	2, 0, 0	0, 2, 2	0.05	0.05
3	1	1, 0, 0	1, 2, 2	0.2	0.2
4	1	0, 0, 0	0, 2, 2	0.3	0.2
5	2	0, 0, 0	4, 4, 4	0.5	0.5
6	2	4, 0, 0	0, 4, 4	0.003	0.002
7	2	2, 0, 0	2, 4, 4	0.1	0.1
8	2	3, 0, 0	1, 4, 4	0.03	0.02
9	2	1, 0, 0	3, 4, 4	0.2	0.2

## Appendix D: Operating characteristics

Operating characteristics for different design specifications are provided in Table 3. Fixed Balanced Randomised and Bayesian Response-Adaptive designs are as reported in the Protocol. Under Mapped designs, we have considered complete data (i.e. no missingness in biomarkers at 8 weeks) and missing data cases.

**Table 3:** Summary of designs and their respective statistics under different missing data scenarios

Design	Level of missingness	No. of missing data points	Type I error (under $\varvec{H}_{\varvec{0}}$)	Power (SE) (under $\varvec{H}_{\varvec{1}}$)	Allocation of placebo (SD) (under $\varvec{H}_{\varvec{1}}$)	Allocation (SD) of inferior arm (under $\varvec{H}_{\varvec{1}}$)	Allocation (SD) of superior arm (under $\varvec{H}_{\varvec{1}}$)
Fixed Balanced Randomised Design	Complete data	(0, 0, 0)	0.12	0.75 (0.01)	33 (10)	33 (11)	34 (11)
Bayesian Response-Adaptive Design	Complete data	(0, 0, 0)	0.11	0.79 (0.01)	32 (7)	21 (10)	47 (12)
Mapped Design	Complete data	(0, 0, 0)	0.11	0.80 (0.01)	30 (0)	20 (11)	50 (11)
	Case 1	(1, 0, 0)	0.11	0.76 (0.01)	30 (2)	20 (12)	50 (12)
	Case 2	(2, 0, 0)	0.10	0.75 (0.02)	30 (3)	20 (13)	50 (13)
	Case 3	(0, 1, 0)	0.11	0.76 (0.01)	30 (2)	20 (12)	50 (12)
	Case 4	(0, 2, 0)	0.11	0.74 (0.01)	30 (3)	20 (12)	50 (13)
	Case 5	(1, 1, 0)	0.10	0.74 (0.01)	30 (4)	20 (13)	50 (13)

## Appendix E: Rationale—updating randomisation

Update 1 is at the end of randomising 6 + 3 patients from blocks 1 and 2. The possible allocations for stage 2 are 2:1:3 or 2:3:1 or 2:2:2 for $C: T_1 : T2$. Therefore, even if we allocate the first three patients as balanced, it is still difficult to predict the remaining assignments as well as we can easily switch to favouring an arm if required after the second patient of stage 2. Additionally, we also expect the first interim analysis to conclude by the first update point. Thus, we save considerable time by not stopping for the interim analysis.

Update 2 is at the time of interim 2. Stage 3 allows dropping and without knowledge of the interim analysis results, any pre-specified allocation would only contain placebo to allow the possibility of dropping. Therefore, to maintain unpredictability, the sequence is updated during the interim itself if needed. Also, the second interim analysis could be quicker as we have already performed one before so the experience gained could speed up the process.

## Appendix F: Rationale—missing data handling (interim)

We perform simulation-based sensitivity analyses considering missing data in stages 1 and 2 to decide whether to deviate from balanced allocation if suggested by the response-adaptive design. We do not consider any missingness in stage 3 for both the strata, since this is the final stage of the trial and will not influence any adaptation decisions. All simulations are carried out under a missing-at-random setting, where missingness occurs randomly in one of the arms without any underlying structure with the associated outcome or patient characteristics.

To evaluate the performance of the trial with missing data, we compute by the operating characteristics containing power, type I error and patient benefits, and compare these metrics with those of the complete (no-missing data) scenario as presented in [13].

*Interim 1 decision:* We find that the favour % is very high under the null hypothesis which is undesirable since we want to be conservative in terms of adapting during missingness. To mitigate this, we choose not to adapt and maintain balanced allocation of 2:2:2 for stage 2.

*Interim 2 decision:* This follows similar reasoning as the previous point, where under the null, we observe high chances of dropping an arm which is not desired. However, we will allow favouring an arm if the data suggest so.

## Appendix G: Simulation details for pooled data analysis

A pooled hypothesis testing analysis, combining data across strata, will be performed as a secondary analysis when active treatments show a similar direction of the estimated treatment effect, formally defined by its sign: $\text {sign}(\widehat{ATE}^A_{k}) = \text {sign}(\widehat{ATE}^B_{k})$ for each $k = T1, T2$. The decision rule is based on simulation studies in which we have evaluated the operating characteristics (type I error and power) of a *stand-alone* analysis (i.e. independently by stratum, each based on $n=20$) vs. a pooled analysis (based on $N=40$).

Specifically, we considered nine scenarios that varied both in direction and magnitude of the treatment effect; these are reported in Table 4. We considered three main cases: strata with identical treatment effects (identical strata), strata with treatment effects slightly varying in their magnitude but not in their direction (similar strata), strata with treatment effects varying in their direction (divergent strata). Figure 4 shows the frequentist operating characteristics of a pooled type of analysis in these cases, compared to a *stand-alone* one. Overall, we can see that scenarios S2–S4 greatly benefit from a pooled analysis, with substantial power improvements at a small type I error inflation cost (depicted in scenario S1, which represents the global null). The greatest advantages are encountered in scenarios with treatment effects that are identical but too small to be detected with the trial sample (e.g. scenario S3, where the treatment effect of $T_1$ is 0.2). Similar strata (scenarios S5 and S6) also show a power improvement, although to a smaller extent. However, in divergent strata (scenarios S7–S9), in addition to a potential negative impact on power, a pooled analysis can also affect interpretation. In S9, for example, where the true treatment effect is 0 in exactly one of the two strata, a pooled analysis leads to a $75\%$ probability of rejecting the null of no treatment effect for both $k=T_1$ and $k=T_2$. Therefore, cases in which the two treatments show a divergent mechanism of action in the two strata will not be evaluated with a pooled analysis.

**Table 4:** Simulation scenarios with specification of the “true” treatment effects per stratum: $\mathbb {E}\Delta Y_{k} - \mathbb {E}\Delta Y_{C}$, for $k = T_1, T_2$

	Stratum A			Stratum B		
Scenario	$\varvec{\mathbb {E}\Delta Y}_{\varvec{C}}$	$\varvec{\mathbb {E}\Delta Y}_{\varvec{T}_{\varvec{1}}}$	$\varvec{\mathbb {E}\Delta Y}_{\varvec{T}_{\varvec{2}}}$	$\varvec{\mathbb {E}\Delta Y}_{\varvec{C}}$	$\varvec{\mathbb {E}\Delta Y}_{\varvec{T}_{\varvec{1}}}$	$\varvec{\mathbb {E}\Delta Y}_{\varvec{T}_{\varvec{2}}}$
Scenario S1: identical strata (global null)	0.0	0.0	0.0	0.0	0.0	0.0
Scenario S2: identical strata	0.0	0.0	0.3	0.0	0.0	0.3
Scenario S3: identical strata	0.0	0.2	0.3	0.0	0.2	0.3
Scenario S4: identical strata	0.0	0.3	0.4	0.0	0.3	0.4
Scenario S5: similar strata	0.0	0.0	**0.2**	0.0	0.0	**0.3**
Scenario S6: similar strata	0.0	0.0	**0.3**	0.0	0.0	**0.4**
Scenario S7: divergent strata	0.0	0.0	**0.0**	0.0	0.0	**0.3**
Scenario S8: divergent strata	0.0	**0.0**	**0.3**	0.0	**0.3**	**0.3**
Scenario S9: divergent strata	0.0	**0.3**	**0.0**	0.0	**0.0**	**0.3**

The bold font highlights the difference in the nonidentical scenarios

## Appendix H: SAP checklist

Here is the reporting checklist [17] for the SAP.

**Table 5:** Statistical Analysis Plan (SAP) Checklist v 1.0 2019

Section/Item	Index	Description	Reported on page #
**Section 1: Administrative information**			
Trial and Trial registration	1a	Descriptive title that matches the protocol, with SAP either as a forerunner or subtitle, and trial acronym (if applicable)	1
	1b	Trial registration number	1
SAP Version	2	SAP version number with dates	12
Protocol Version	3	Reference to version of protocol being used	2
SAP revisions	4a	SAP revision history	12
	4b	Justification for each SAP revision	12
	4c	Timing of SAP revisions in relation to interim analyses, etc.	12
Roles and responsibility	5	Names, affiliations, and roles of SAP contributors	10
Signatures of:	6a	Person writing the SAP	10
	6b	Senior statistician responsible	10
	6c	Chief investigator/clinical lead	10
**Section 2: Introduction**			
Background and rationale	7	Synopsis of trial background and rationale including a brief description of research question and brief justification for undertaking the trial	1
Objectives	8	Description of specific objectives or hypotheses	2
**Section 3: Study Methods**			
Trial design	9	Brief description of trial design including type of trial (e.g., parallel group, multi-arm, crossover, factorial) and allocation ratio and may include brief description of interventions	2
Randomization	10	Randomization details, e.g., whether any minimization or stratification occurred (including stratifying factors used or the location of that information if it is not held within the SAP)	5, 6
Sample size	11	Full sample size calculation or reference to sample size calculation in protocol (instead of replication in SAP)	4
Framework	12	Superiority, equivalence, or noninferiority hypothesis testing framework, including which comparisons will be presented on this basis	8
Statistical interim analysis and stopping guidance	13a	Information on interim analyses specifying what interim analyses will be carried out and listing of time points	4
	13b	Any planned adjustment of the significance level due to interim analysis	4
	13c	Details of guidelines for stopping the trial early	7
Timing of final analysis	14	Timing of final analysis, e.g., all outcomes analysed collectively or timing stratified by planned length of follow-up	4
Timing of outcome assessments	15	Time points at which the outcomes are measured including visit “windows”	3
**Section 4: Statistical Principals**			
Confidence intervals and *P* values	16	Level of statistical significance	4
	17	Description and rationale for any adjustment for multiplicity and, if so, detailing how the type 1 error is to be controlled	4
	18	Confidence intervals to be reported	4
Adherence and Protocol deviations	19a	Definition of adherence to the intervention and how this is assessed including extent of exposure	NA
	19b	Description of how adherence to the intervention will be presented	NA
	19c	Definition of protocol deviations for the trial	NA
	19d	Description of which protocol deviations will be summarized	NA
	20	Definition of analysis populations, e.g., intention to treat, per protocol, complete case, safety	4
**Section 5: Trial Population**			
Screening data	21	Reporting of screening data (if collected) to describe representativeness of trial sample	7
Eligibility	22	Summary of eligibility criteria	4
Recruitment	23	Information to be included in the CONSORT flow diagram	NA
Withdrawal/Follow-up	24a	Level of withdrawal, e.g., from intervention and/or from follow-up	NA
	24b	Timing of withdrawal/lost to follow-up data	NA
	24c	Reasons and details of how withdrawal/lost to follow-up data will be presented	NA
Baseline patient characteristics	25a	List of baseline characteristics to be summarized	7
	25b	Details of how baseline characteristics will be descriptively summarized	7
**Section 6: Analysis**			
Outcome definitions		List and describe each primary and secondary outcome including details of:	3
	26a	Specification of outcomes and timings. If applicable include the order of importance of primary or key secondary end points (e.g., order in which they will be tested)	3
	26b	Specific measurement and units (e.g., glucose control, hbA1c [mmol/mol or %])	3
	26c	Any calculation or transformation used to derive the outcome (e.g., change from baseline, QoL score, Time to event, logarithm, etc.)	3
Analysis methods	27a	What analysis method will be used and how the treatment effects will be presented	8
	27b	Any adjustment for covariates	9
	27c	Methods used for assumptions to be checked for statistical methods	9
	27d	Details of alternative methods to be used if distributional assumptions do not hold, e.g., normality, proportional hazards, etc.	9
	27e	Any planned sensitivity analyses for each outcome where applicable	9
	27f	Any planned subgroup analyses for each outcome including how subgroups are defined	9
	28	Reporting and assumptions/statistical methods to handle missing data (e.g., multiple imputation)	9
	29	Details of any additional statistical analyses required, e.g., complier-average causal effect10 analysis	9
	30	Sufficient detail on summarizing safety data, e.g., information on severity, expectedness, and causality; details of how adverse events are coded or categorized; how adverse event data will be analysed, i.e., grade 3/4 only, incidence case analysis, intervention emergent analysis	9
Statistical software	31	Details of statistical packages to be used to carry out analyses	5
References	32a	References to be provided for nonstandard statistical methods	5, 8
	32b	Reference to Data Management Plan	NA
	32c	Reference to the Trial Master File and Statistical Master File	NA
	32d	Reference to other standard operating procedures or documents to be adhered to	NA

**Taken from the paper:** Gamble C, Krishan A, Stocken D, Lewis S, Juszczak E, Doré C, et al. Guidelines for the Content of Statistical Analysis Plans in Clinical Trials. JAMA. 2017;318(23):2337-43

**Abbreviations:** *CONSORT* Consolidated Standards of Reporting Trials, *hbA1c* haemoglobin A1c, *QoL* quality of life, *SAP* statistical analysis plan

*The development of this checklist was funded by the MRC Hubs for Trials Methodology Research*
